# Validation of housekeeping gene and impact on normalized gene expression in clear cell Renal Cell Carcinoma: critical reassessment of YBX3/ZONAB/CSDA expression

**DOI:** 10.1186/1471-2199-15-9

**Published:** 2014-05-16

**Authors:** Sébastien Dupasquier, Anne-Sophie Delmarcelle, Etienne Marbaix, Jean-Pierre Cosyns, Pierre J Courtoy, Christophe E Pierreux

**Affiliations:** 1 CELL Unit, de Duve Institute and Université catholique de Louvain UCL-ICP, Avenue Hippocrate 75, 1200 Brussels, Belgium; 2 Pathology Department, Cliniques universitaires St.-Luc and Université catholique de Louvain, Avenue Hippocrate 10, 1200 Brussels, Belgium

## Abstract

**Background:**

YBX3/ZONAB/CSDA is an epithelial-specific transcription factor acting in the density-based switch between proliferation and differentiation. Our laboratory reported overexpression of YBX3 in clear cell renal cell arcinoma (ccRCC), as part of a wide study of YBX3 regulation in vitro and in vivo. The preliminary data was limited to 5 cases, of which only 3 could be compared to paired normal tissue, and beta-Actin was used as sole reference to normalize gene expression. We thus decided to re-evaluate YBX3 expression by real-time-PCR in a larger panel of ccRCC samples, and their paired healthy tissue, with special attention on experimental biases such as inter-individual variations, primer specificity, and reference gene for normalization.

**Results:**

Gene expression was measured by RT-qPCR in 16 ccRCC samples, each compared to corresponding healthy tissue to minimize inter-individual variations. Eight potential housekeeping genes were evaluated for expression level and stability among the 16-paired samples. Among tested housekeeping genes, PPIA and RPS13, especially in combination, proved best suitable to normalize gene expression in ccRCC tissues as compared to classical reference genes such as beta-Actin, GAPDH, 18S or B2M. Using this pair as reference, YBX3 expression level among a collection of 16 ccRCC tumors was not significantly increased as compared to normal adjacent tissues. However, stratification according to Fuhrman grade disclosed higher YBX3 expression levels in low-grade tumors and lower in high-grade tumors. Immunoperoxidase confirmed homogeneous nuclear staining for YBX3 in low-grade but revealed nuclear heterogeneity in high-grade tumors.

**Conclusions:**

This paper underlines that special attention to reference gene products in the design of real-time PCR analysis of tumoral tissue is crucial to avoid misleading conclusions.

Furthermore, we found that *global* YBX3/ZONAB/CSDA *mRNA* expression level may be considered within a “signature” of RCC grading.

## Background

Comparison of gene expression levels between individuals and/or biological or pathological conditions requires internal reference such as so-called housekeeping genes (HKGs), so that normalized expression can correct for variations in amounts of starting RNA and/or minimize biases due to reverse transcription efficiency. The ideal HKG(s) selected for normalization should not be influenced by experimental or biological conditions to be compared; in particular, its own expression level should be identical in tumors and adjacent healthy sites. Frequently, reports focus on a gene of interest (GOI) and the reference gene is assumed to remain stable without validation. However, a growing number of studies showed that the expression levels of many commonly used HKGs are affected by experimental conditions or vary in pathological states, particularly in cancer, and thus stressed the risk of blind use of classical HKGs [1-4]. For instance, despite wide use, GAPDH and beta-Actin should no longer be considered suitable as internal references in so diverse conditions such as cell proliferation, differentiation, metabolic changes, hypoxia and cancer [5-11]. Since no universal reference has yet been discovered, the choice of HKG(s) should be validated in each condition studied. To this aim, bioinformatics tools such as NormFinder, BestKeeper and GeNorm have been recently developed [12-14].

Y-box protein 3 or YBX3 (approved HGNC name symbol), also known as CSDA (Cold Shock Domain protein A), ZONAB (zonula occludens 1 (ZO-1)-associated nucleic acid binding protein) or dbpA (DNA binding protein A), is a multifaceted epithelial-specific protein able to (i) act as a transcription factor, (ii) regulate mRNA stability and (iii) interact with and regulate function of other proteins. YBX3 is thus implicated in the regulation of epithelial morphogenesis and homeostasis by modulating multiple cellular processes such as the control of cell density, proliferation, differentiation, or survival. For instance, YBX3 interacts with the cell cycle regulating kinase CDK4, and upregulates PCNA (Proliferative Cell Nuclear Antigen) and Cyclin-D1 gene transcription [15,16]. In kidney proximal tubular epithelial cells, YBX3/ZONAB/CSDA not only stimulates proliferation, but also represses genes involved in apical differentiation such as cubilin and megalin/LRP-2 (low density lipoprotein receptor-related protein 2) [17]. More recently, YBX3 binding to the p21 mRNA was shown to stabilize and enhance p21 mRNA translation, thereby promoting cell survival in response to cellular stress [18]. Other studies have implicated YBX3 in tumor development. Although YBX3 alone is not sufficient to induce liver tumor development [19], its overexpression and nuclear localization in hepatocarcinomas have been correlated to poorer prognosis [20-22]. YBX3 up-regulation has also been reported to play a role in the pathogenesis of gastric cancer, increasing cell invasion and tumor chemoresistance [23]. In opposition, YBX3 would display anti-oncogenic effects in squamous cell carcinomas, inhibiting tumor growth and metastasis [24].

Clear cell renal cell carcinomas (ccRCC) rank among ten most frequent cases of cancer death in developed countries because of its bad prognosis, linked to a high propensity to metastasize. Our laboratory recently explored the expression level of YBX3 in a limited number of non-graded ccRCC as part of a wide study of YBX3/ZONAB regulation in vitro and in vivo [17]. Using beta-actin as reference, YBX3 was found to be overexpressed, although the group of Jung demonstrated that beta-actin was not appropriate for ccRCC studies [25]. Moreover, the only other study of YBX3 expression in ccRCC reported only modest overexpression in only one out of ten tumor cases studied [26].

We thus here re-evaluated the expression level of YBX3 as well as other genes implicated in ccRCC in a larger panel of ccRCC samples (16 instead of 5 previously used), each compared to adjacent non tumoral tissue, and evaluated 8 different HKGs to select for best reference. We confirmed by multiple analysis that beta-Actin is not appropriate as internal reference for studies of gene expression in ccRCC but found that combination of two HKGs, PPIA (coding for peptidylprolyl isomerase A) and RPS13 (coding for the ribosomal protein S13), minimizes fluctuations when comparing ccRCC samples to their adjacent healthy tissues. Based on this combined reference, we found no global difference in YBX3 expression in ccRCC when analyzed all together, as compared to normal tissue. However, stratification of ccRCC samples according to tumor grade revealed that global YBX3/ZONAB/CSDA expression level is higher in the low-grade tumors and lower in the high-grade tumors.

## Methods

### Samples

The 16 ccRCC tumor samples and their matched healthy tissues used in this publication were provided by the UCL Biolibrary at the Cancer Centre of the Cliniques Universitaires Saint-Luc (http://www.centreducancer.be/en/show/index/section/8/page/34), Brussels, Belgium, project #CDCUCLR, approved by the BioMedical Ethics Committee of the Université catholique de Louvain. All tissue samples are provided by the Biolibrary according to the Belgian law, with the informed consent of the patients and full agreement from the BioMedical Ethics Committee of the Université catholique de Louvain. Samples and data are coded anonymously. Patients’ tumor clinical features are compiled in Table 1.

**Table 1:** Patient tumors casuistics

**Tumor number**	**Gender**	**Age**	**TNM classification**			**Grade**	**Relevant anteriorities**	**% Tumoral**	**% Non tumoral**
			**pT**	**pN**	**cM/pM**				
1	M	66	pT3b	pNx	cM0	1	No	90	10 - fibrosis
2	M	77	pT3a	pN0	pM1	2	No	100	
3	M	50	pT3a	pNx	cM1	3	No	100	
4	M	53	pT1b	pNx	cM0	2	No	90	10 - fibrosis
5	M	43	pT1b	pNx	cM0	3	No	50	50 - hemorraghes, fibrosis, macrophages
6	M	64	pT3a	pNx	cM0	2	No	75	25 - fibrosis
7	M	58	pT3a	pNx	cM0	1	No	60	40 - fibro-vascular
8	M	60	pT1b	pNx	cM0	1	No	100	
9	F	59	pT2b	pN0	cM0	2	No	70	30 - fibrosis
10	M	45	pT1b	pNx	cM0	2	No	95	5 - fibrosis
11	M	74	pT3a	pNx	cM0	2	No	100	
12	M	61	pT3a	pNx	cM0	3	No	100	
13	M	57	pT2a (m)	pN0	pM1	3	No	60	40 - normal parenchyma
14	F	71	pT1a	pNx	cM0	2	Yes*	100	
15	F	62	pT1a	pNx	cM0	1	No	100	
16	M	66	pT3a	pN0	cM0	3	No	60	40 - fibrosis

*, Primary ccRCC of the left kidney 19 years ago, treated by surgery. Other ccRCC of the right kidney and metastasis in the thyroid 7 years ago, treated by surgery. Metastasis in the pancreas 3 years ago, treated by surgery. Lobular carcinoma of the right breast treated by mastectomy, axillary dissection, radiotherapy and tamoxifen 3 years ago.

Two experimented senior pathologists (EM and JPC) independently checked all tissues for diagnosis confirmation, percentage of tumor cells and tumor grading. All tumor samples sections were found to be mainly composed of tumor cells (see Table 1); all “normal” paired samples were tumor-free, with variable fibrosis.

### Total RNA isolation and reverse transcription

Several cryostat slices (cumulative thickness ≈ 100 μm) of OCT-embedded frozen tissue were pooled and homogenized in 500 μl of TriZol (Invitrogen), followed by addition of 30 μg glycogen. Lysates were transferred on phase-Lock gel (5Prime) to improve phase separation and to avoid DNA contamination. 100 μl chloroform was added, samples were vigorously shaken then centrifuged at 4°C (12000 *g* for 15 min). The upper phase was transferred into a fresh tube and RNA was precipitated with isopropanol (2:1) at -20°C overnight, followed by 15 min centrifugation as above. RNA pellets were washed in 75% ethanol and finally resuspended in 30 μl RNAse/DNAse-free water. Concentration was determined in a Nanodrop spectrophotometer and the quality of RNA was controlled by 260/280 and 260/230 absorbance ratios (ratios were comprised between 1.84 and 1.95, and between 1.65 and 2.14, respectively). For reverse transcription, 1 μg of total RNAs in 10 μl RNAse/DNAse-free water was first denaturated by heating at 85°C for 5 min, followed by rapid cooling on ice. 10 μl containing 200 units MMLV reverse transcriptase with buffer, 200 ng of primer random p(dN)_6_ oligonucleotides, 500 μM dNTP, 10 mM DTT, and 40 units of RNAse OUT (final concentrations; all from Invitrogen, except random oligonucleotides from Roche) were then added to denatured RNA. The mixture was incubated successively at 25°C for 15 min, 37°C for 1 h, and 75°C for 10 min. Finally, cDNA was diluted to a final concentration of 10 ng of starting RNA per μl and stored at -20°C.

### Real-time qPCR

RTqPCR experiments were performed in 10 μl containing 1 μl of cDNA, 200nM of gene specific primers and 5 μl of 2x Kapa SYBR fast qPCR master mix (KapaBiosystems). White 96 wells plates (BioRad) were used on a BioRad CFX96 thermocycler.

Cycling conditions were as follows: an initial step at 95°C 5 min for enzyme activation, followed by 45 cycles alternation of 3 sec at 95°C; 30 sec at 60°C and a final dissociation step.

Primers used are listed in Table 2. Primers for YBX3/CSDA (that we here call YBX3 + Ψ), CycD1, 18S ribosomal RNA and beta-Actin have been published [17,27]. Primers for PCNA were from RTPrimerDB database (http://medgen.ugent.be/rtprimerdb/). For megalin/LRP-2 and Cubilin we resorted to TaqMan probes (Cat. # 4331182 and # 4331182 Life technologies). The other targets were designed using NCBI primerblast (http://www.ncbi.nlm.nih.gov/tools/primer-blast). Only primer pairs located in different exons separated by an intronic sequence of at least 1000 bp were considered. Specificity was verified (i) *in silico*, by Blast analysis on *homo sapiens* Refseq RNA (taking into account not only validated mRNAs but all including hypothetical RNAs); and (ii) experimentally, based on a single dissociation peak in melting curve analysis of cDNA samples and absence of amplification in negative controls (10 ng of non retro-transcribed RNA; H_2_O). Amplicon sizes were checked on agarose gel and PCR efficiency was assayed for all primer sets by serial dilutions of cDNA and reported in Table 1 according to Rasmussen formula: E = 10^-1/slope^[28]. As all primer sets exhibited a comparable efficiency around 2 (100%), the 2^-ΔΔCt^ formula, where ΔCt = Ct_GOI_ – Ct_HKG_, and ΔΔCt = ΔCt_Tumor_ – ΔCt_Normal,_ was used to calculate the fold-expression in tumours compared to matched normal tissue [29,30]. When the pair of HKGs (i.e. PPIA + RPS13) was used to normalize gene expression, Ct_HKG_ in the above formula was the geometric mean of the two Ct values. To homogenize runs, the PCR threshold used to determine Ct was systematically set at 1000RFU, i.e. at the middle of the logarithmic phase of SYBR incorporation. For each primer set, variability was assessed in 3 to 5 independent PCR runs; mean variations were inferior to 2% except for 18S, which reached 4.5%.

**Table 2:** List and characteristics of primers used in real-time PCR experiments

**Target**	**Full name [mRNA NCBI accession ID]**	**Primer sequence (5′ to 3′)**	**Amplicon size (bp)**	**Slope**	**r**^**2**^	**Eff**
**β-Act**	Actin beta [NM_001101.3]	F: AGGCCAACCGCGAGAAGATGACC	332	-3.486	0.998	1.94
		R: GAAGTCCAGGGCGACGTAGCAC				
**GAPDH**	glyceraldehyde-3-phosphate dehydrogenase [NM_002046.4]	F: TTCTTTTGCGTCGCCAGCCGA	96	-3.402	0.998	1.97
		R: GTGACCAGGCGCCCAATACGA				
**18S**	18S ribosomal RNA [NR_003286.2]	F: GGCGCCCCCTCGATGCTCTTAG	89	-3.488	1	1.94
		R: GCTCGGGCCTGCTTTGAACACTCT				
**B2M**	beta-2-microglobulin [NM_004048.2]	F: TGCCTGCCGTGTGAACCATGT	97	-3.319	0.997	2.00
		R: TGCGGCATCTTCAAACCTCCATGA				
**RP2**	polymerase (RNA) II (DNA directed) polypeptide A [NM_000937.4]	F: TCCCATGGGTGGAATCTCTCCTGC	162	-3.767	0.995	1.84
		R: GAGTAACCTGGGCTGAAGCCGC				
**PPIA**	peptidylprolyl isomerase A (cyclophilin A) [NM_021130.3]	F: ACCGCCGAGGAAAACCGTGTA	129	-3.217	0.999	2.05
		R: TGCTGTCTTTGGGACCTTGTCTGC				
**RPL27**	ribosomal protein L27 [NM_000988.3]	F: TGGTAGGGCCGGGTGGTTGC	185	-3.203	0.997	2.05
		R: ACTTTGCGGGGGTAGCGGTC				
**RPS13**	ribosomal protein S13 [NM_001017.2]	F: TCGGCTTTACCCTATCGACGCAG	153	-3.429	0.999	1.96
		R: ACGTACTTGTGCAACACCATGTGA				
**YBX3 +** **Ψ**	Y box binding protein 3 [NM_003651.4/NM_001145426.1]	F: C**GG**TTCATCGAAATCCAACT	166	-3.519	0.999	1.92
		R: TAATTGTAGGGACGCCGGTA				
**YBX3-Ψ**	Y box binding protein 3 [NM_003651.4/NM_001145426.1]	F: CCACCAAAGTCCTTGGCACTGTC	240	-3.452	0.998	1.95
		R: T**C**CC**T**TCCACAGGAACTCCA**T**CC**G**				
**VEGFa**	vascular endothelial growth factor A [NM_001025366.2]	F: AGAAACCACGCTGCCGCCAC	118	-3.191	0.993	2.06
		R : GTCTCGCCCTCCGGACCCAA				
**c-Myc**	v-myc myelocytomatosis viral oncogene homolog [NM_002467.4]	F: TACAACACCCGAGCAAGGAC	189	-3.2	0.998	2.05
		R: AGCTAACGTTGAGGGGCATC				
**CycD1**	cyclin D1 [NM_053056.2]	F: CGCCCCACCCCTCCAG	221	-3.136	0.997	2.08
		R: CCGCCCAGACCCTCAGACT				
**PCNA**	proliferating cell nuclear antigen [NM_002592.2]	F: GTAGTAAAGATGCCTTCTGGTG	190	-3.425	0.997	1.96
		R: TCTCTATGGTAACAGCTTCCTC				

Database source for sequence design is the NCBI gene browser (http://www.ncbi.nlm.nih.gov/gene). PCR efficiency was calculated with standard curves (slope and r^2^ included) according to Rasmussen formula [28]. (F: Forward; R: Reverse). Note that bases indicated in bold text in YBX3 primers correspond to mismatches in the YBX3 pseudogene 1 sequence [NR_027011.1].

### Primers design for YBX3

For detection of YBX3 mRNA, two primer pairs were used. Primers used previously [17] are here called “YBX3 + Ψ” (where Ψ is its known pseudogene), as two independent *in silico* PCR programs (University of California Santa Cruz (UCSC) Genome Browser [31]. NCBI primer blast) revealed that these primers co-amplified YBX3 mRNA but also that of a pseudogene (YBX3P1, Gene ID: 440359, updated on 8-Apr-2014) located on human chromosome 16. Accordingly, a new pair of primers specific to the authentic gene, named “YBX3-Ψ”, was designed with Primer-blast, using same criteria as above. We found that the sense “YBX3-Ψ” primer hybridized perfectly to YBX3 and YBX3P1, while the antisense harbored four bases that differ from YBX3P1, two at the 3′ end and two at the 5′ end (Table 2). Specificity was confirmed by *in silico* analysis; however despite the 4 divergent bases in the antisense primer, YBX3-Ψ primers could amplify in vitro the YBX3P1 pseudogene from genomic DNA. We tested the efficiency of the two YBX3 primer pairs on serial dilutions of genomic DNA, from 1 ng to 15 pg (equivalent to 10% to 0.15% contamination of the RNA preparation). In that range the amplification efficiency with YBX3-Ψ primers was reduced as compared to the amplification with YBX3 + Ψ primers: 82% *vs* 96%.

### Immunohistochemistry

Cryostat slices (≈10 μm) of OCT-embedded frozen tissue were stained with the anti-YBX3 antibody (IBL; #18981) according to the protocol described (22). Images were acquired using a Zeiss Mirax Midi microscope.

### Statistics

Statistical analyses were performed using GraphPad Prism (version 5.00; GraphPad Software, San Diego CA). We selected the non-parametric Wilcoxon test for paired data to compare HKG levels and normalized gene expression levels between tumor and healthy tissues. The non-parametric MannWhitney test was used to compare YBX3 expression between graded tumor groups. Correlation coefficients were calculated using Spearman rank method. For all analyses, a p < 0.05 was considered as statistically significant.

To determine HKG stability, we used the RefFinder free online access (http://www.leonxie.com/referencegene.php). RefFinder integrates the currently available major computational programs (Normfinder [12], BestKeeper [13], geNorm [14] and the comparative ΔCt method [32]) to compare and to rank the candidate reference genes.

## Results and discussion

### Expression and stability of HKG in ccRCC

To optimize normalization of target genes expression in ccRCC, we measured the expression levels of 8 candidate HKGs in a panel of 16 ccRCC and paired non-malignant tissue. We compared levels of the widely used 18S ribosomal RNA, beta-Actin (β-Act), glyceraldehyde-3-phosphate dehydrogenase (GAPDH) and beta-2-microglobulin (B2M) mRNAs, as well as mRNAs for the less classical RNA polymerase type II (RP2) and peptidylprolyl isomerase A (PPIA), and the recently described potential HKGs ribosomal protein L27 (RPL27) and ribosomal protein S13 (RPS13) [33].

**Figure 1:**
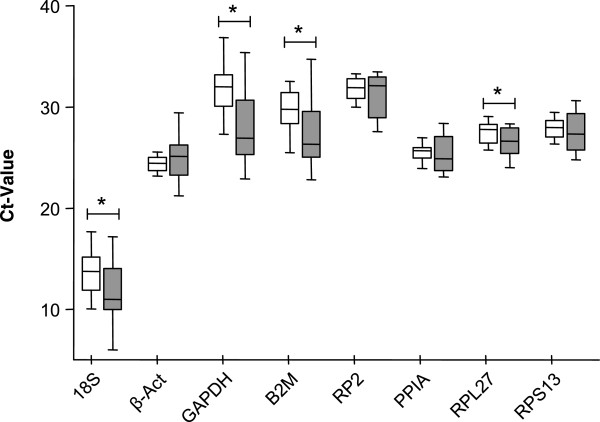
**Comparison of expression level for the 8 indicated housekeeping genes (HKG) in the 16 ccRCC samples (grey boxes) and their paired normal tissue (open boxes).** Values are cycle threshold (Ct) cross-points as defined in Material and Methods. Boxes are from lower to upper quartiles intersected by medians; whiskers are Min/Max values in the cohort of samples. GAPDH, 18S, B2M and RPL27 significantly differed between paired control versus tumoral groups (*p < 0.05 by Wilcoxon Test, see Table 3).

Results were first analyzed using standard statistical tools. Figure 1 shows raw Ct values obtained in all tumors (grey) and normal samples (white), presented as box and whiskers plots. As expected, values for 18S ribosomal RNA (mean Ct 12.6) were lower than mean Ct values of the seven other HKGs tested (between 24.7 and 31.4). Since Ct values for GAPDH, 18S, B2M, and RPL27 RNAs were significantly different between tumor and normal samples (p < 0.05, Wilcoxon test), these should be a priori disqualified to normalize gene expression in ccRCC samples (* in Figure 1 and Table 3). In contrast, the mean expression levels of the four other mRNAs tested, RP2, β-Act, PPIA and RPS13 did not vary significantly between the two groups (p > 0.05, Wilcoxon test). Among these, PPIA and RPS13 exhibited the most comparable Ct values between tumor and healthy tissues (p = 0.626 and p = 0.715 respectively, Figure 1 and Table 3). The stability of the potential HKG was also studied by calculating the coefficient of variation of raw Ct (CtCV) obtained in each group (normal: intraN; and tumor: intraT), and in all the biological samples whatever their diseased state (Tot) (Table 3). Globally, the expression levels of all the HKGs tested were less stable in tumor samples (CtCV > 5% in intraT) than in normal samples (CtCV < 5% in intraN, except for 18S, GAPDH and B2M). The larger variability of HKG among tumor samples as compared to normal tissues from which they arise underline the fact that each tumor is unique and may exhibit genomic instability. The coefficient of variation of each HKG in all biological samples taken as a whole (CtCV Tot) was not dependent on the level of expression or Ct values (see Figure 1) but correlated to their Wilcoxon p value testing statistical difference between normal and tumoral groups. These variations illustrate the differences that do exist among individuals. These observations underscore the need to work with matched tissues and to critically select the best HKG in order to minimize biases when assaying gene expression.

**Table 3:** Evaluation by conventional statistical analysis of HKG levels and stability

	**Wilcoxon p value**	**CtCV intraN (%)**	**CtCV intraT (%)**	**CtCV Tot (%)**
**18S**	0.042*	15.94	29.15	24.52
**β-Act**	0.391	3.34	9.11	6.85
**GAPDH**	0.017*	7.71	13.13	12.72
**B2M**	0.042*	6.82	12.54	10.96
**RP2**	0.194	3.59	6.59	5.45
**PPIA**	0.626	3.58	6.66	5.36
**RPL27**	0.035*	3.97	7.06	6.26
**RPS13**	0.715	3.55	7.56	5.90

Statistical differences between Ct for tumoral versus normal tissue were assessed by non-parametric paired Wilcoxon test. Highest p values indicate best candidates; *(p < 0.05) indicates unsuitable HKG. Coefficients of variation (CtCV) are given for normal (intraN), tumoral (intraT) and all confounded (Tot) samples. Lower values reflect lesser dispersion and better stability.

Altogether, this standard statistical analysis disclosed that PPIA and RPS13 displayed the most comparable and stable expression levels between normal and tumor samples, thereby best qualified as internal reference in ccRCC samples.

This conclusion, and the respective values of the other RNAs used for normalization, were further tested using dedicated algorithms available online. RefFinder (http://www.leonxie.com/referencegene.php?type=reference; see Methods section) is a user-friendly web-based comprehensive tool developed to evaluate and screen reference genes from large experimental datasets. It integrates the currently available major computational programs, geNorm, Normfinder, BestKeeper, and the comparative ΔCt method, to compare and rank the candidate reference genes according to their dispersion as a stability score [12-14,32]. A gene with a low score has a stable expression and will thus be better ranked. Based on ranking in each of these programs, RefFinder assigns an appropriate score to each individual HKG and calculates the geometric mean of their scores in a final ranking. As this *in silico* tool only considers stability of gene levels, but not different biological populations, it is comparable to the CtCV analysis (Table 3). Thus, when comparing several experimental conditions (e.g. tumor *vs* normal) it should be used in combination with inter-group statistical analysis as above.

Table 4 summarizes rankings obtained with the different algorithms for the eight HKGs. This *in silico* analysis on the entire (normal and tumoral) cohort of samples confirmed that PPIA and RPS13 mRNAs belong to the most stable housekeeping gene product tested. Interestingly, beta-Actin mRNA was less stable: except with BestKeeper, it was scored in the second half of the four other rankings. The GeNorm algorithm not only supported best rankings of PPIA and RPS13 evidenced with the other algorithms, but also provides search for best combinations of HKGs, which, not surprisingly, was PPIA with RPS13 (Table 4). Although RPL27 could also be considered as a good HKG in terms of stability based on this program, with an identical stability score as PPIA (Table 4), paired analysis revealed that its level varied statistically between tumors and healthy tissues (Figure 1 and Table 3).

**Table 4:** Ranking of HKG stability by public algorithms

**Ranking**	**Ref finder**	**Ge norm**	**Norm finder**	**Best keeper**	**delta Ct**
**1**	RPS13 (1.68)	PPIA/RPS13 (0.600)	RPL27 (0.244)	PPIA (1.074)	RPL27 (1.82)
**2**	PPIA (1.86)	RPL27 (0.883)	RPS13 (1.252)	RPS13 (1.202)	RPS13 (1.97)
**3**	RPL27 (1.86)	RP2 (1.038)	RP2 (1.276)	b-Act (1.250)	PPIA (2.01)
**4**	RP2 (3.94)	b-Act (1.206)	PPIA (1.387)	RPL27 (1.286)	RP2 (2.07)
**5**	b-Act (5.01)	18S (1.776)	18S (1.885)	RP2 (1.520)	18S (2.51)
**6**	18S (5.48)	B2M (2.084)	B2M (2.198)	18S (2.367)	b-Act (2.52)
**7**	B2M (6.74)	GAPDH (2.343)	b-Act (2.206)	B2M (2.472)	B2M (2.72)
**8**	GAPDH (8.00)		GAPDH (2.844)	GAPDH (3.161)	GAPDH (3.12)

Each HKG (or pair of HKG) is ranked according to the indicated algorithms (respective stability scores indicated in brackets) and RefFinder assigned a final rank (geometric mean of stability scores for each gene). Lower values indicate more stable genes. Note that (i) PPIA and RPS13 rank at the top of 3 out of 5 algorithms; (ii) GAPDH and B2M are worst candidates; and (iii) beta-Actin ranks intermediate.

These results and analysis of candidate HKGs in ccRCC thus supported and extended the study of Jung [25], which already warned against using beta-Actin mRNA as only internal reference to normalize RT-qPCR results in ccRCC, and proposed PPIA as alternative. Our analysis identified RPS13 as an alternative good HKG for ccRCC. Interestingly, the GeNorm algorithm indicated that RPS13 and PPIA together provide the best combination of tested HKGs for gene expression studies in ccRCC. We propose that both approaches (inter-group statistical analysis and *in silico* global stability determination) could be advantageously used to identify the most reliable HKG or combination thereof.

In the second part of this study, we thus used the objectively selected combination of PPIA and RPS13 as reference genes to normalize that of other genes in ccRCC samples.

### Critical evaluation of YBX3/ZONAB/CSDA expression in ccRCC

Using this selected combination of HKG mRNAs, we thus re-evaluated the expression of YBX3 in 16 ccRCC tumor samples and paired normal tissue, including the 3 samples of our previous study for which normal tissue was available [17].

YBX3 gene (NCBI Gene ID: 8531) is located on chromosome 12 at locus p13.1 and encodes two spliced mRNA isoforms (NCBI mRNA accession IDs: NM_003651.4 and NM_001145426.1). However, human genome also contains a pseudogene for YBX3, YBX3P1, on chromosome 16 [34] (NCBI Gene ID: 440359, updated on 8-Apr-2014). This intron-less sequence is almost identical to YBX3 transcript (95% identity in coding region) and could introduce a bias in mRNA quantification through genomic DNA contamination, but also if this pseudogene is transcriptionally active [35-37]. To avoid this possibility, we designed a second pair of primers aimed at distinguishing cDNA derived from authentic YBX3 mRNA *vs* its pseudogene. The first pair, used previously [17], was noted YBX3 + Ψ, because two independent *in silico* PCR programs (UCSC Genome Browser; NCBI primer blast) showed that these co-amplify YBX3 mRNA and its pseudogene. The second pair of primers, named YBX3-Ψ, was designed to be specific for authentic YBX3 transcript only. Although the same *in silico* PCR analysis confirmed its specificity, in vitro experiments on genomic DNA revealed that YBX3-Ψ pair could amplify the pseudogene from genomic DNA (see Methods). However, at low - or real - contaminating genomic DNA concentration, YBX3-Ψ primers amplified the pseudogene with a reduced efficiency as compared to YBX3 + Ψ. We thus preferred (and recommend the use of) the YBX3-Ψ primers pair.

**Figure 2:**
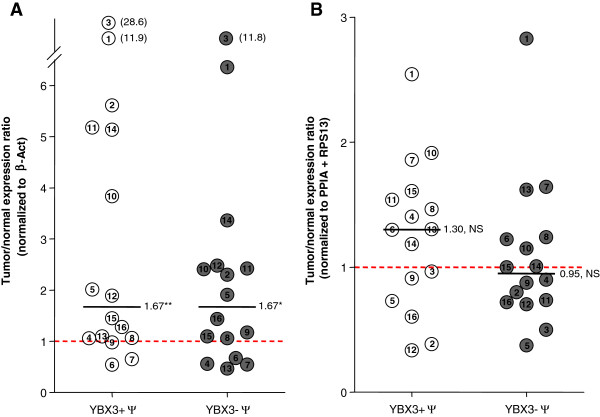
**Effects of primer choice of reference HKG on the estimation of normalized YBX3 expression in ccRCC samples.** Circles represent expression ratios distribution of YBX3 mRNA (YBX3-Ψ, filled circles; YBX3 + Ψ, open circles) in each of the 16 tumors compared to its normal paired tissue (set at 1, dot line) after normalization by beta-Actin **(A)** versus PPIA + RPS13 **(B)**. Values are means of 5 independent assays (for the sake of clarity, standard deviations and statistical significance for each sample are not shown, but are provided in the histograms of Additional file 2). Note that pairs 1 to 3 were analyzed in the previous report [17]. Lines are medians with indicated values. After normalization by beta-Actin, YBX3 appears significantly overexpressed in tumors taken as a whole **(A**, **p < 0.01, *p < 0.05, Wilcoxon test) while it is not the case when normalized by PPIA and RPS13 **(B**, NS: not significant, Wilcoxon test).

Figure 2 shows YBX3 expression as dot plot. Expression of YBX3+/- Ψ in all 16 pairs of samples was first normalized to beta-Actin (Figure 2A) in order to compare with results from our previous study [17], then to the geometric mean of the optimal combination of selected HKGs for ccRCC identified above, PPIA and RPS13 (Figure 2B). The relative YBX3 mRNA expression in tumors was presented as ratio to the corresponding value in adjacent non-tumoral tissue, set as the unity. In most cases, there was little difference in YBX3 quantification when comparing the two pairs of primers (compare open and filled circles).

When expression of YBX3 was normalized to that of β-Act, global analysis of the 16 samples revealed a wider distribution and a significant 1.67-fold YBX3 overexpression in tumors, yet limited to about half samples and at a modest, if not marginal level. Of the three samples used in the previous study [17], sample #1 (x6.4) and #3 (x11.8) showed the highest apparent overexpression of YBX3 when normalized to beta-Actin. However, when normalized to the geometric mean of PPIA and RPS13, these samples display a 2.8- and 0.5-fold expression. This illustrates the strong impact of the choice of HKG to normalize gene expression, and the need to carefully validate this internal reference a priori. Moreover, it emphasizes the need to re-evaluate YBX3 expression in ccRCC.

When the geometric mean of PPIA and RPS13 expression levels was used to normalize authentic YBX3 expression in the 16 samples, the distribution was less dispersed and this cohort no longer showed a significant difference in YBX3 expression in tumor samples, as compared to matched controls (p = 0.9780; Wilcoxon test), in agreement with the report by Kohno and colleagues [26].

### Expression of other ccRCC-associated genes

To ensure that the combination of HKGs selected for normalization of gene expression in ccRCC did not introduce an opposite bias (i.e. by damping individual differences), we further analyzed the expression of key genes known to be implicated in the pathogenesis of, and overexpressed in, ccRCC: VEGFa, cMyc and Cyclin-D1.

**Figure 3:**
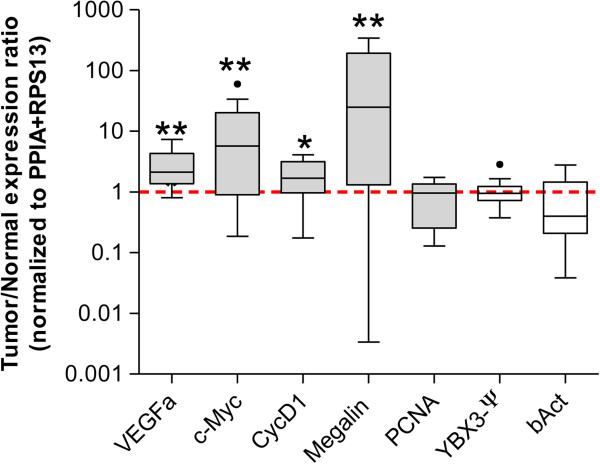
**Expression ratios of other cancer-related genes normalized to PPIA and RPS13 housekeeping genes.** Boxes represent the lower and upper quartiles with medians in the 16 tumor samples compared to paired normal tissues (set at 1, red dot line); whiskers indicate the Tukey confidence intervals and black dots (•) are outlier values. VEGFa, c-Myc, CycD1 and megalin are significantly overexpressed in tumors (***p < 0.001, **p < 0.01, *p < 0.05, Wilcoxon Test) in comparison to PCNA and YBX3, which are globally not different from paired normal tissues. Beta-Actin expression is variable in tumor samples with a tendency to be down-regulated. (Please note the logarithmic Y axis due to strong c-Myc and Megalin overexpression).

The most prevalent cause of ccRCC (80% of patients) is inactivation of von Hippel-Lindau (VHL) gene, by allelic deletions, mutations, or epigenetic silencing [38-40]. Inactivation of VHL results into stabilization of Hypoxia Inducible Factors (HIF) and their accumulation in the nucleus, thereby inducing a panel of genes including VEGFa (Vascular Endothelial Growth Factor), PDGF (Platelet-Derived Growth Factor), EGF (Epidermal Growth Factor), and TGF (Transforming Growth Factor) that ultimately lead to neo-angiogenesis and tumor progression [41,42]. Blocking the VEGF pathway indeed emerged as a promising therapeutic strategy in ccRCC [43,44]. Even if less studied than the VHL/HIF/VEGFa pathway, the c-Myc proto-oncogene and CyclinD1 have also been clearly implicated in kidney tumorigenesis. As is the case in many other cancers, c-Myc is overexpressed in a majority of clear cell renal tumors [45,46], sometimes associated with c-Myc gene locus amplification [47-49], and is essential for enhanced proliferation of ccRCC tumor cells [46]. The pro-proliferative CyclinD1 is a c-Myc target and is highly expressed in 50-75% ccRCC cases [50-52]. Moreover, the VHL pathway has been reported to control c-Myc [53,54] and CyclinD1 [55,56].We thus measured the expression of VEGFa, c-Myc and CyclinD1 in the 16 tissue pairs and normalized their expression to that of the geometric mean of PPIA and RPS13 (Figure 3). Tumor samples clearly exhibited a significant overexpression of VEGFa (***p = 0.0009), c-Myc (**p = 0.0041) and CyclinD1 (*p = 0.0162), as compared to their matched normal tissues (Figure 3, Wilcoxon test). Normalization with the combined references genes thus confirmed published data, indicating they did not introduce masking bias.

### Expression of YBX3 target genes

We then assessed the expression of known targets of YBX3/CSDA/ZONAB in the ccRCC samples and studied the correlation of their expression with that of YBX3. CyclinD1 and proliferative cell nuclear antigen (PCNA) contain YBX3-responsive elements in their promoters and are stimulated by YBX3 (16). On the contrary, megalin and cubilin genes also contain “CCAAT” boxes in their regulatory regions, but in these cases YBX3 binding represses transcription (17).

In the 16 tumor tissues, CyclinD1 expression was stimulated as compared to their paired samples (Figure 3). Interestingly, variations of CyclinD1 expression in the cohort correlated with YBX3 expression (Additional file 1): low YBX3-expressing tumors had low levels of CyclinD1, while high YBX3 tumors displayed high CyclinD1 mRNAs. PCNA expression level in the ccRCC tumors was not different from normal tissues and accordingly no correlation was found with YBX3 (Figure 3 and Additional file 1). Cubilin, which is repressed by YBX3 in vitro, was discarded from our analysis as we could not reproducibly amplify cubilin mRNA in all the 16 paired samples. Megalin/LRP-2 has been shown by microarray and RT-qPCR to be overexpressed in clear cell as well as in papillary RCC [57]. This observation was confirmed in our 16 tumor samples that exhibited a significant overexpression of megalin/LRP-2 as compared to their paired controls (Figure 3; **p = 0.0041). Correlation of megalin expression with YBX3 showed a trend to an inverse relationship but did not reach statistical significance (Additional file 1). This correlation study suggests that in ccRCC, YBX3 level of expression may affect CyclinD1 expression and may also participate in megalin/LRP-2 expression.

Finally, we re-analysed beta-Actin, not only as a classical HKG, but also as a gene per se based on its normalized expression by reference to combined PPIA and RPS13. As already observed in Figure 1, β-Act expression in the 16 tumor tissues was variable and exhibited a trend to be globally down-regulated in ccRCC samples, even if not reaching statistical significance (Figure 3). This observation again argues against the use of beta-Actin as a reference when measuring gene expression in ccRCC, and requires one to reconsider YBX3 overexpression in most cases of our previous study [17].

Intriguingly, some reports suggest a possible role of actin expression in the tumorigenesis process. Actin is a major component of cytoskeleton, and altered cell morphology is a characteristic of tumor cells, notably to enable invasiveness, migration and metastasis. For example, accumulation of beta-actin in tips of pseudopodia drives invasiveness and metastatic ability of transformed MDCK cells [58]. Indeed, even though mechanisms are not yet well understood, changes in expression level of beta-Actin were associated with higher invasiveness of hepatoma cells [59], and with metastatic potential of colon adenocarcinoma cell lines [60]. Besides its direct role in the control of cell morphology, actin dynamics could also regulate genetic programs by the so-called mechanogenetic mechanisms [61,62]. Indeed, ablation of beta-Actin altered the ratio of globular actin to filamentous actin in mouse embryonic fibroblasts, with corresponding changes in expression of genes regulating cell cycle and motility [63].

Interestingly, except for one obvious outlier, variations of beta-Actin expression in the cohort we analyzed appeared to correlate with YBX3 expression (Additional file 1). Variations of beta-Actin expression also followed in parallel YBX3 expression during mouse kidney embryonic development (data not shown). Based on the well-known roles of YBX3 in the control of epithelium morphogenesis, one can hypothesize that YBX3 could regulate beta-Actin expression during kidney ontogenesis and oncogenesis.

### Expression of YBX3/ZONAB/CSDA as a function of tumor grade

**Figure 4:**
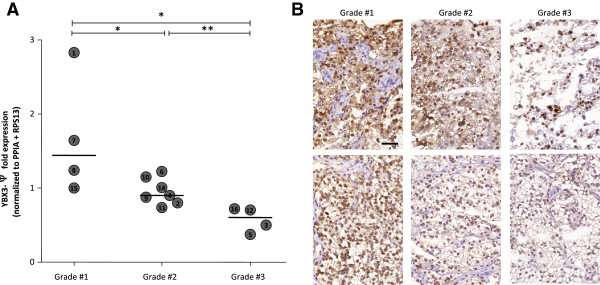
**Distribution of YBX3 expression ratios in tumor samples according to histological grading. (A)** Circles are values for YBX3-Ψ expression ratios in the 15 Fuhrman-graded tumors compared to paired adjacent healthy tissue after normalization to geometric mean of PPIA + RPS13. Lines are medians for each group and statistical differences between subgroups are indicated as well (Mann Whitney test, *p < 0.05, **p < 0.01). The expression of YBX3 inversely correlates with tumor grade. **(B)** Representative images of YBX3 protein staining in tumor samples. Note (i) absence of nuclear labeling in stromal nuclei; (ii) overall decrease of intensity from grade #1 to #3; (iii) occasional cytoplasmic labeling in grade #2; and (iv) marked heterogeneity of nuclear labeling in some fields of grade #3 only. Scale bar; 50 μm.

Although we concluded above that YBX3 did not show systematic overexpression in the global cohort of ccRCC samples, a closer look suggested the possibility of three expression groups: high, intermediate, and low. Indeed, as compared to matched normal tissues, five tumors (# 1, 6, 7, 8, 13) exhibited a significant overexpression of YBX3; five other (# 3, 5, 11, 12, 16) exhibited significant underexpression; while the six remaining (# 2, 4, 9, 10, 14, 15) did not show significant difference from their normal counterparts (Figure 2B, filled circles, YBX3-Ψ values normalized to PPIA and RPS13; and Additional file 2). We thus wondered if these variations in YBX3 levels could be related to ccRCC pathogenesis and more specifically to tumor grades and asked two experimented pathologists to independently grade the 16 tumor samples according to Fuhrman (nuclear appearance), being totally unaware of our expression level data. Both pathologists agreed on the 16 cases; one case was nevertheless excluded from further analysis as it contained 40% of normal parenchyma (see Table 1). Of the 15 remaining cases 4 tumor samples were graded #1, 7 were graded #2 and 4 were graded #3. Segmentation of YBX3 expression levels according to histopathological grading is shown in Figure 4. This analysis revealed that YBX3 negatively correlates to tumor grade with higher expression levels in grade #1, intermediate in grade #2, and lower in grade #3 (Figure 4 and additional file 3). This correlation was further analyzed at the protein level by immunohistology in tumor samples from grade #1 to #3 (Figure 4B). YBX3 staining was clearly evident in all nuclei of grade #1 tumors. In grade #2 tumors, YBX3 staining was weaker in the nuclei and sometimes appeared in the cytoplasm. In grade #3 tumors, we observed strong inter- and intra-tumor heterogeneity of YBX3 staining; varying from complete absence to intense staining in some nuclei. These qualitative histopathological observations are in agreement with our RT-qPCR analyses showing decreased expression level of YBX3 from grade #1 to grade #2 and 3 (compare panel A and B of Figure 4).

Even if these numbers were small, the negative correlation of YBX3 mRNA expression level with tumor anaplasia, hence potential progression, was clearly significant. Upregulation of YBX3 in grade #1 tumors, followed by progressive silencing in more anaplastic, hence more prone to metastasize, could indicate that YBX3 plays a biological role at a low degree of neoplastic transformation characterizing tumors with low metastatic potential, and be dispensable for disease progression. A similar trend has been recently reported in hepatocellular carcinoma (HCC). Although YBX3 overexpression and nuclear localization was initially correlated to poor patients outcome [22], a recent study by the same team revealed that high YBX3 level in pre-cancerous “hypercarcinogenic” states, but not in established HCC, was, in fact, associated to poor prognosis [64]. It is thus conceivable that high YBX3 expression would promote dedifferentiation and proliferation, thus secondarily favor mutations of cancer as a multistep process [65,66]. An interesting alternative hypothesis is based on the ability of YBX3 to reduce tumor angiogenesis and lymphangiogenesis, so as to inhibit tumor growth and metastasis in lung cancer and squamous cell carcinoma models [24,67]. The authors went so far as to propose YBX3 as a potential “therapeutic” gene, which, if artificially induced in tumors, could prevent disease progression and dissemination. Of note, YBX3 is also down-regulated in breast cancer, as compared to healthy tissue [68]. However, the grade of the breast cancer was not mentioned. If this interpretation is correct, YBX3 extinction in most anaplastic, hence potentially invasive tumor cells, would promote angio/lympho-genesis, thus favor metastatic dissemination.

## Conclusion

In conclusion, although the pathogenic implications of YBX3 in (renal) human tumorigenesis clearly need to be further explored, its global mRNA expression level may be part of a “signature” of RCC grading.

## Competing interests

The authors declare that no conflict of interest exists.

## Authors’ contributions

SD and ASD conceived, designed, performed the experiments, analyzed results and drafted the manuscript. EM and J-PC graded tumor samples and helped to draft the manuscript. PJC analyzed results and drafted the manuscript. CEP supervised the study, analyzed the results and drafted the manuscript. All authors read and approved the final manuscript.

## Supplementary Material

Additional file 1 **YBX3 correlation with known target genes and beta-Actin.** Dot plots with respective mean fold variations of YBX3-Ψ (X axis) and CyclD1 (A), PCNA (B), megalin/LRP-2 (C) and B-Act (D) (Y axis) in the 16 tumor samples compared to adjacent healthy tissues. Red dotted lines indicate values in adjacent healthy tissues. Black lines show linear regression curves. Please note the logarithmic scale for megalin expression. Spearman coefficient of correlation and p value are indicated. Note that value of sample1 (open circle) can be considered as outlier (outside Tukey confidence interval) and was excluded. Click here for file

Additional file 2 **Effects of primer and choice of reference HKGs on the estimation of normalized YBX3 expression in ccRCC samples.** Expression of YBX3 mRNA, measured with YBX3 + Ψ (open bars) and YBX3-Ψ (filled bars) primers, in each of the 16 tumors compared to adjacent healthy tissue (set at 1, red dotted line) after normalization to either beta-Actin (**A**), or PPIA & RPS13 (**B**). Values are means of 5 independent assays with standard deviations. Tumor values statistically different from their normal paired tissues are indicated by asterisks (*). Pairs 1 to 3 were those analyzed in our previous report [17]. Click here for file

Additional file 3 **Distribution of YBX3** **+** **Ψ expression ratios in tumor samples according to histological grading.** Circles are values for YBX3 + Ψ expression ratios in the 15 Fuhrman-graded tumors compared to paired adjacent healthy tissue after normalization to geometric mean of PPIA + RPS13. Lines are medians for each group and statistical differences between subgroups are indicated as well (Mann Whitney test, *p < 0.05, NS, not significant). The expression of YBX3 inversely correlates with tumor grade. Click here for file
