# Supplementary figures and images for: Validation of housekeeping gene and impact on normalized gene expression in clear cell Renal Cell Carcinoma: critical reassessment of YBX3/ZONAB/CSDA expression
Source: BMC Mol Biol. 2014 May 16;15:9. doi: 10.1186/1471-2199-15-9 (PMC4045873; doi:10.1186/1471-2199-15-9)

**A**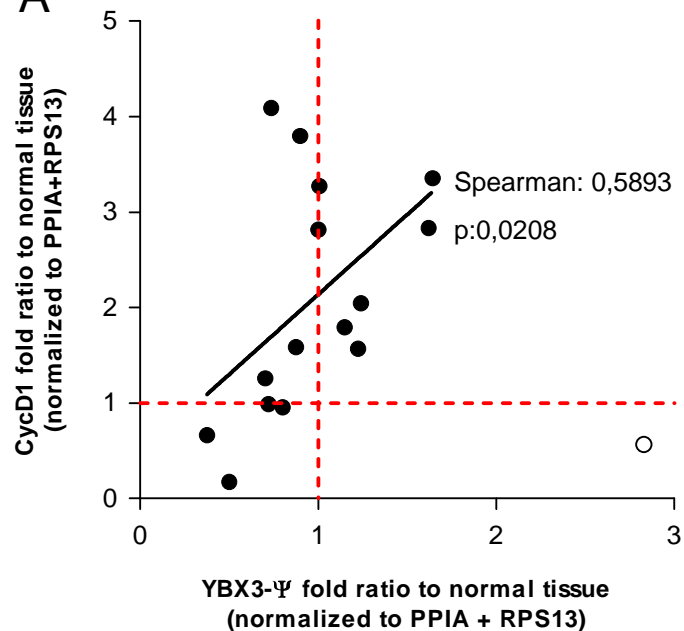**C**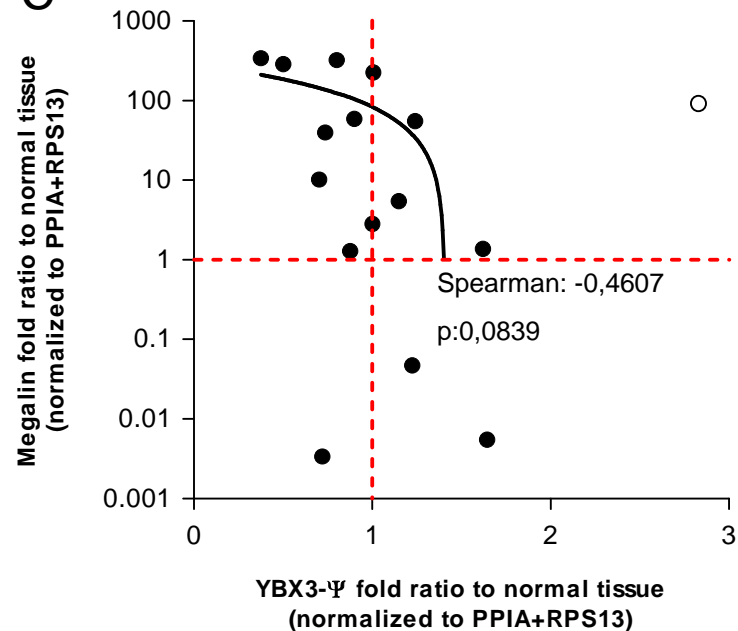**B**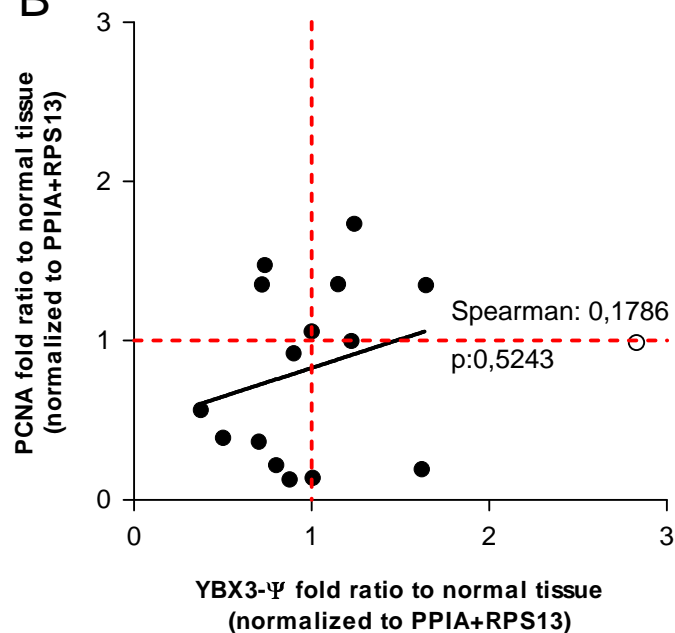**D**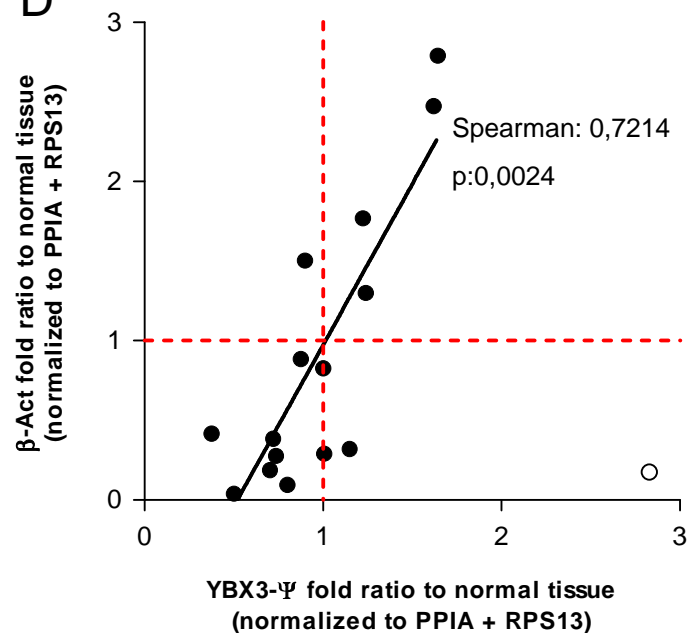

Supplement: Additional file 1 — YBX3 correlation with known target genes and beta-Actin. Dot plots with respective mean fold variations of YBX3-Ψ (X axis) and CyclD1 (A), PCNA (B), megalin/LRP-2 (C) and B-Act (D) (Y axis) in the 16 tumor samples compared to adjacent healthy tissues. Red dotted lines indicate values in adjacent healthy tissues. Black lines show linear regression curves. Please note the logarithmic scale for megalin expression. Spearman coefficient of correlation and p value are indicated. Note that value of sample1 (open circle) can be considered as outlier (outside Tukey confidence interval) and was excluded. [file 1471-2199-15-9-S1.pdf]

**A**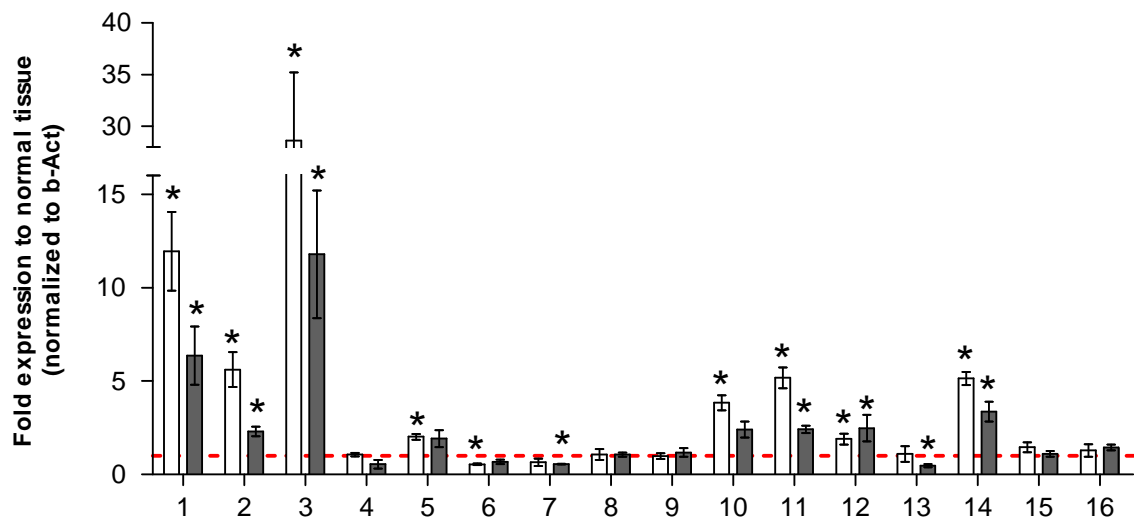**B**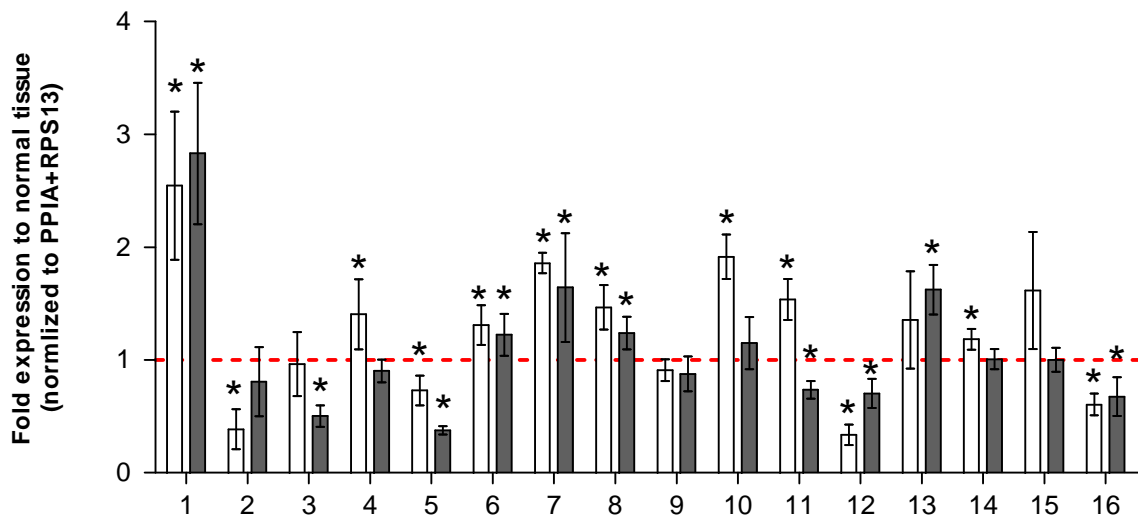

Supplement: Additional file 2 — Effects of primer and choice of reference HKGs on the estimation of normalized YBX3 expression in ccRCC samples. Expression of YBX3 mRNA, measured with YBX3 + Ψ (open bars) and YBX3-Ψ (filled bars) primers, in each of the 16 tumors compared to adjacent healthy tissue (set at 1, red dotted line) after normalization to either beta-Actin (A), or PPIA & RPS13 (B). Values are means of 5 independent assays with standard deviations. Tumor values statistically different from their normal paired tissues are indicated by asterisks (*). Pairs 1 to 3 were those analyzed in our previous report [17]. [file 1471-2199-15-9-S2.pdf]

YBX3+ $\Psi$  fold expression  
(normalized to PPIA + RPS13)

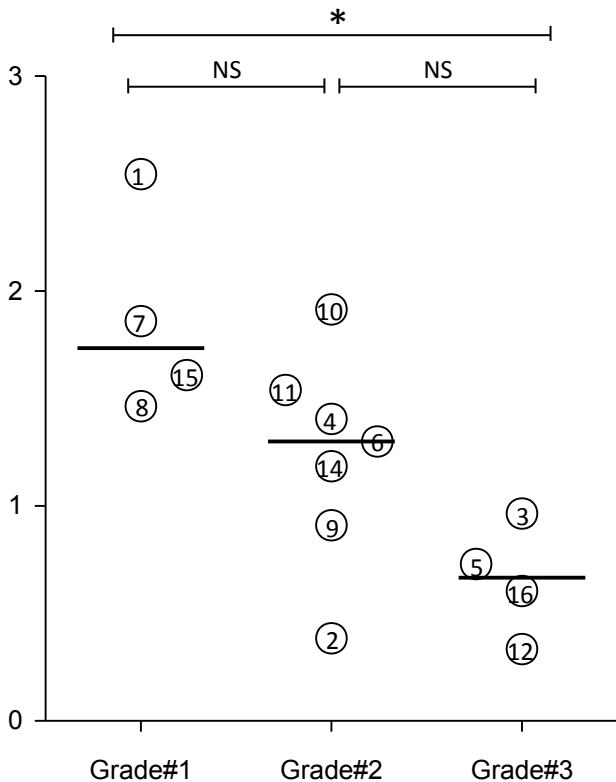

Supplement: Additional file 3 — Distribution of YBX3 + Ψ expression ratios in tumor samples according to histological grading. Circles are values for YBX3 + Ψ expression ratios in the 15 Fuhrman-graded tumors compared to paired adjacent healthy tissue after normalization to geometric mean of PPIA + RPS13. Lines are medians for each group and statistical differences between subgroups are indicated as well (Mann Whitney test, *p < 0.05, NS, not significant). The expression of YBX3 inversely correlates with tumor grade. [file 1471-2199-15-9-S3.pdf]
